# How Does Stress Lead to Risk of Alcohol Relapse?

**DOI:** 10.35946/arcr.v34.4.07

**Published:** 2012

**Authors:** Rajita Sinha

**Affiliations:** **Rajita Sinha, Ph.D.,***is professor of psychiatry and child study, Yale University School of Medicine, New Haven, Connecticut.*

**Keywords:** Alcoholism, alcohol dependence, alcohol and other drug (AOD)-seeking behavior, AOD craving, alcohol cue, relapse, relapse prevention, recovery, motivation, risk factors, stress, stress response, brain, brain imaging, biomarker, intervention, human studies

## Abstract

Empirical findings from human laboratory and brain-imaging studies are consistent with clinical observations and indicate that chronic alcohol-related dysfunction in emotional and stress responses plays a role in motivation to consume alcohol in people with alcohol use disorders. Recent findings on differences in stress responsivity in alcohol-dependent versus nondependent social drinkers demonstrate alterations in stress pathways that partially may explain the significant contribution of stress-related mechanisms on craving and relapse susceptibility. These findings have significant implications for clinical practice, including (1) the development of novel brain and stress biology–related measures of relapse risk that could serve as biomarkers to identify those most at risk of alcohol relapse during early recovery from alcoholism; and (2) the development of novel interventions that target stress-related effects on the motivation to drink alcohol and on relapse outcomese

It has long been known that stress increases the risk of alcohol relapse (Sinha 2001). Clinical observations, surveys, and epidemiological studies document an association between self-reports of stressors and subsequent return to drinking. Studies assessing alcohol relapse after treatment completion and discharge also indicate the contribution of highly stressful events independent of alcohol use history that increase the risk of subsequent relapse (Brown et al. 1990). Furthermore, negative mood and stress are associated with increased craving, and high levels of urges to use alcohol predict relapse (Cooney et al. 2007). However, the mechanisms by which stress exposure increases alcohol relapse risk have been elusive, until recently. The last two decades have seen a dramatic increase in preclinical and clinical research to understand psychobiological and neural evidence linking stress and alcohol consumption. Evidence suggests that the neural circuits involved in stress and emotions overlap substantially with the brain systems involved in drug reward. Chronic alcohol use can result in neuroadaptive changes in stress and reward pathways. Such changes may alter an alcohol-dependent person’s response to stress, particularly with respect to stress and emotion regulation and motivation for alcohol, which in turn may increase the risk of relapse (Sinha 2001, 2005).

To put the stress and alcohol relapse linkage in the clinical context, the sidebar presents sample descriptions of an acute stressful life event and an acute alcohol-related situation that led to subsequent alcohol use in a person with alcohol dependence. The patient vignettes are descriptions provided by patients currently in treatment and refer to previous experiences and episodes of alcohol use and relapse.

## Chronic Alcohol-Related Changes in Emotion, Stress, and Motivational Systems

Converging lines of evidence indicate that regular and chronic alcohol use is associated with changes in emotion, stress, and motivational pathways. These changes may in turn influence alcohol craving and relapse risk. Chronic alcohol use increases stress-related symptoms and is associated with increased anxiety and negative emotions; changes in sleep and appetite; aggressive behaviors; changes in attention, concentration, and memory; and desire/craving for alcohol (Sinha 2001, 2007, 2009). Stress-related symptoms are most prominent during early abstinence from chronic alcohol use, but some of these changes also have been documented during active use of specific drugs. Chronic alcohol abuse and acute alcohol withdrawal states are associated with heightened activity in the brain stress systems, such as increased secretion of the stress hormones corticotropin-releasing factor (CRF), norepinephrine, and cortisol in a number of the brain’s stress and emotion centers, such as the hypothalamus^1^, amygdala, hippocampus, and prefrontal regions (Koob and Kreek 2007). Chronic alcohol abuse also alters dopaminergic signaling in the ventral striatum (VS) and the ventral tegmental area (VTA). And such changes are associated with increased alcohol seeking (craving) and alcohol self-administration in laboratory animals (Cleck and Blendy 2008; Koob and Kreek 2007; Koob et al. 2004; Rasmussen et al. 2006). Further corroboration from human neuroimaging studies indicates that chronic alcohol abuse reduces dopamine receptors (i.e., D2 receptors) in striatal regions and dopamine transmission in the frontal lobe in alcoholics during acute withdrawal and protracted withdrawal (up to 3–4 months) (see Volkow 2004 for review). Functional imaging studies indicate increased VS activity in response to alcohol cues and altered brain response in the amygdala to emotional stimuli with chronic alcohol use (Gilman and Hommer 2008; Heinz et al. 2004, 2005; Martinez et al. 2007).

The biological stress response is most commonly detected in humans by activation of the hypothalamic–pituitary–adrenal (HPA) axis involving CRF-stimulated release of adrenocorticotropin (ACTH) from the anterior pituitary, which in turn stimulates the adrenal glands to release the stress hormone cortisol, which is involved in mobilizing and regulating the body’s stress response. The second pathway involved in the biological stress response is the autonomic nervous system, comprising the sympathetic and the parasympathetic components. The sympathetic component mobilizes arousal by increasing heart rate and blood pressure; the parasympathetic component enforces the “brakes” for sympathetic arousal and functions to decrease and regulate autonomic function. Alcohol use stimulates the HPA axis and initially stimulates the autonomic systems by provoking sympathetic arousal, followed by depressing such activation (Ehrenreich et al. 1997; Lee and Rivier 1997). Reductions in this alcohol-related HPA axis response (similar to tolerance) has been demonstrated with regular and chronic alcohol abuse in animals (Lee and Rivier 1997; Richardson et al. 2008; Zhou et al. 2000) and in humans (Adinoff et al. 1998, 2005; Wand and Dobs 1991).

Likewise, chronic alcohol abuse increases physiological arousal as measured by heart rate but also decreases heart rate variability, which serves as a measure of parasympathetic function (Ingjaldsson et al. 2003; Rechlin et al. 1996; Shively et al. 2007; Thayer and Sternberg 2006). These data represent alcohol-induced changes in peripheral stress pathways, which parallel basic science findings of alcohol-related adaptations in central stress systems, namely the extrahypothalamic CRF and the noradrenergic pathways that are indicative of hyperresponsive brain stress pathways noted in the previous paragraph (Cleck and Blendy 2008; Koob and Kreek 2007; Koob 2009; Rasmussen et al. 2006). These neurochemical changes indicate specific dysregulation in the neurochemical systems that play a role in emotion, stress, and motivation functions in alcoholics. Such changes raise the question of whether these measures contribute to the high levels of emotional distress, alcohol craving, and compulsive alcohol seeking that may lead to increased relapse susceptibility.

## Effects of Stress on Alcohol Craving and Arousal

Drug craving or “wanting” for drug is a hallmark feature of addiction. It is an important component in maintaining addictive behaviors (Dackis and Gold 1985; O’Brien et al. 1998; Robinson and Berridge 1993, 2000; Tiffany 1990). Chronic alcohol use leads to changes in the brain reward and motivation pathways that can increase alcohol craving in the context of alcohol and alcohol-related stimuli, but also in the context of stress. In support of these ideas, a growing literature indicates that people with alcohol abuse show greater alcohol craving than social drinkers (Glautier et al. 1992; Greeley et al. 1993; Kaplan et al. 1985; Pomerleau et al. 1983; Willner et al. 1998). Furthermore, severity of alcohol use has been shown to affect the magnitude of cue-related physiological arousal, compulsive alcohol seeking, and stress-related changes, including alcohol-related morbidity (Fox et al. 2005; Grusser et al. 2006, 2007; Rosenberg and Mazzola 2007; Sinha 2008; Yoon et al. 2006). These data are consistent with large population-based studies indicating that the risk of alcohol-related problems, addiction, and chronic diseases increases with greater weekly or daily alcohol and drug use (Dawson et al. 2005; Rehm et al. 2009; Room et al. 2005). Given these responses, the author’s research examined whether increases in craving are associated with altered stress responses that occur with chronic alcohol use.

In the clinical context, alcoholic patients entering outpatient substance abuse treatment report high levels of stress and an inability to manage distress adaptively, thereby increasing the risk of succumbing to high levels of drug craving and relapse to drug use (Sinha 2007). Although patients often are successful in learning cognitive–behavioral strategies in treatment, relapse rates remain high (Brandon et al. 2007; Sinha 2011). These data suggest possible difficulties in applying and accessing cognitive–behavioral strategies in real-world relapse situations. Thus, to understand the biobehavioral mechanisms underlying the high stress and craving state during early recovery, the author began to study this phenomenon in the laboratory, using an ecologically relevant method that models such relapse risk. This research used two of the most common relapse situations—emotionally stressful situations and alcohol-/drug-related situations—in order to develop a comparable method of provoking stress and the drug-related craving state, and these are compared to a relaxing situation that serves as an experimental control condition to account for the nonspecific aspects of the experimental procedures (Sinha 2009).

## Provoking Relapse Situations and Inducing Alcohol and Drug Craving in the Laboratory

To assess relapse risk in laboratory studies, Sinha and O’Malley (1999) targeted alcohol and drug craving as a primary outcome measure that is both a common feature of alcoholism and substance abuse and also is known to relate to the disease state (i.e., high amounts of alcohol use and abuse). The researchers initially compared a commonly used standard social stress task (i.e., giving a speech in front of a video camera with the potential for a monetary reward) with 5-minute individualized guided imagery exposure of each participants’ own recent stressful scenarios. In addicted individuals, stress imagery elicited multiple emotions of fear, sadness, and anger when compared with the stress of public speaking, which elicited increased fear, but no anger and sadness. In addition, individualized stress imagery resulted in significant increases in drug craving, whereas public speaking did not (Sinha and O’Malley 1999).

Another study examined stress-induced and drug-related craving and physiological responses using individualized scripts of comparable length and style for stress, drug- related, and neutral-related situations. Among cocaine-dependent individuals, the imagery exposure to stress and nonstress drug cues resulted in significant increases in heart rate, salivary cortisol levels, drug craving, and subjective anxiety, compared with neutral-relaxing cues (Sinha et al. 2000). Using these methods, researchers have been able to reliably induce alcohol and drug craving in multiple groups of treatment-engaged cocaine-, alcohol-, and opiate-dependent individuals and also increase the desire for the drug in healthy social drinkers (Chaplin et al. 2008; Fox et al. 2007; Hyman et al. 2007; Sinha et al., 2003; see Sinha 2009 for review). In addition, mild to moderate levels of physiological arousal and subjective levels of distress were found to accompany the alcohol/drug craving state (Sinha 2009).

**Patient Vignettes**

These patient descriptions illustrate several points about stress and motivation for alcohol use that are relevant from a clinical perspective. The first vignette is an example of an interpersonal stress situation that is a typical precipitant of relapse. Although patients are less likely to divulge specific details of craving situations in a clinical context, the second vignette illustrates that alcohol cues and increased craving states also promote anxiety and stress-related arousal in people who are alcohol dependent. These clinical situations raise many questions about the role of stress in drug seeking and relapse susceptibility. One such question is whether stress and alcohol cues provoke similar drug craving states that may be targeted in treatment. Additional research questions are whether the response to stress and alcohol-related stimuli differs for alcohol-dependent and non–alcohol-dependent people and whether stress responses and managing stress is altered as a function of chronic alcohol use. These vignettes provide anecdotal evidence; research is needed to address the question of whether craving and stress-related arousal are predictive of relapse outcomes and whether stress causes relapse. Finally, if stress plays an important role in both stress- and cue-related relapse, research is needed to identify the most beneficial types of interventions and how clinicians might use the stress and craving responses to better address the treatment needs of alcohol-dependent individuals in early recovery. The main article addresses each of these questions to elucidate how stress increases the risk of alcohol relapse.

**Stressful Situation**

This situation was rated as a 10 on a 10-point scale of “0 = not at all stressful,” to “10 = highly stressful—most you’ve felt recently” and was narrated by an alcohol-dependent male patient who had been in recovery for 5 weeks. The patient is describing a stressful event that previously led to a relapse episode and an alcohol-related context that led to alcohol use.

“I remember it was about 4:00 pm in the afternoon when Kay woke me up. Her face was red—she looked really upset. She was holding the phone in her hand. She was screaming that I have to call home. I felt tight all over. My heart was pounding. I rolled out of bed. My heart was beating faster. She wants me to call my Dad and tell him about the accident. I did not want to call him yet. She kept following me around the apartment. I tensed up the muscles all over my body. She is badgering me to call. Wherever I go, she was behind me with the phone. I clenched my jaw. I don’t want to face this now, I was thinking. Just call them now and get it over with, she kept saying. My heart was racing. Suddenly, she dialed the number and throws the phone at me while it is ringing. I am gritting my teeth. I put the phone to my ear. My dad answers the phone. I hear his voice. My stomach is in a knot. I start to have a normal conversation. My fists are clenched. I am thinking, “How am I going to tell him about the car accident last night?” I feel jittery and panicky all over. I am pacing back and forth. Casually I say I had a car accident last night. I feel hot all over. He starts screaming, “That’s it! Pack your bags! You’re coming home!” There are butterflies in my stomach. I see Kay burst into tears. I am breathing faster, gasping for air. She is listening to everything he is saying. “What the hell will I tell your mother? I told her you’d be safe. Now I put myself on the line” he is shouting. My head is pounding. Kay is crying, and I can’t do anything about it. I feel stuck. My heart is pounding. My father says he can’t talk anymore now and hangs up the phone. I was so mad, I wanted to smash something. I slam down the phone. I did not want to call him. I knew he would be upset. There is a sinking feeling in my chest. If I could fix it, make it all better, I would. I see Kay crying. I get choked up. I had promised her this would not happen. I feel so mad at myself I want to scream. Now I’ve betrayed her and my Dad.”

**Alcohol-Related Situation**

“It was a bright and sunny summer morning in June. M was gone for the day, and I had the whole day off. I am out working in the yard. It was a warm day and I start to feel hot. I sit down for a break. I’ve done my chores. I’ve paid the bills and vacuumed out the pool. I breathe in deeply. My eyes glance around the yard. I’ve got all the yard work done as well. It looks nice. Now I have half a day left. My heart quickens. I am thinking, ‘is there anything else left to do’. I can’t think of anything else. I feel warm all over. I sit back and try to relax. Now I start feeling very hot. I feel very thirsty. It would be great to have a nice cold beer, I think. I tighten the muscles of my face and forehead. I’ve worked hard, I deserve one, you think. I feel a rush of excitement inside you. I walk inside and head toward the refrigerator. My heart is beating faster. I promised M I won’t drink. My jaws are tight. The thoughts start racing through my head–“She doesn’t need to know.” “She won’t be home for another four hours.” “She won’t be able to smell it on my breath by then.” My hands feel clammy. I open the fridge and grab an ice cold can of beer. My mouth starts to water. Holding that cold can of beer starts to cool down my whole body. I feel a tingling sensation inside me. I start to think—I shouldn’t be drinking this. My stomach is in a knot. I look down at it—it’s right here in my hand, and I deserve it. I wet my lips. Before I know it, I have cracked it open. I see the condensation vapor fly into the air. I can almost taste it now. I am holding on to the can tightly. I raise the can to my lips. I let the beer flow into my mouth and down my throat. It is so cold that it makes my teeth ache. It goes down quickly. I feel a sense of being more alive. Now I have a taste for it. I can’t wait to have another one.”

## Stress Dysregulation and Enhanced Drug Craving in Addicted Individuals

As discussed in the previous section, alcohol-dependent individuals in early recovery show increased stress and alcohol cue–induced craving responses. In a study comparing 4-week abstinent alcoholics with matched social drinkers (drinking less than 25 drinks per month), Sinha and colleagues (2009) found that the recovering alcoholics showed greater levels of basal heart rate and salivary cortisol levels compared with the control drinkers. Upon stress and alcohol cue exposure, they showed greater subjective distress, alcohol craving, and blood pressure responses but blunted stress-induced heart rate and cortisol responses compared with control subjects (Sinha et al. 2009). Furthermore, after exposure to stress imagery, alcoholic patients showed a persistent increase in alcohol craving, subjective distress, and blood pressure responses across multiple time points compared with social drinkers, suggesting an inability to regulate this high alcohol craving and emotional stress state. These data indicate greater allostatic load in abstinent alcoholics, which is accompanied by dysregulated stress responses and high levels of craving or compulsive seeking for the preferred drug.

Together, these data indicate altered stress responses in alcoholics, and these alterations also include an enhanced susceptibility to stress and cue-induced alcohol seeking, which is not seen in healthy nonaddicted individuals. In addition, there are basal alterations in peripheral markers of stress (i.e., stress hormones, such as ACTH and cortisol and in heart rate), indicative of stress-related dysregulation in the CRF–HPA axis and in autonomic responses as measured by basal salivary cortisol and heart rate responses. These high basal responses are associated with lower or blunted stress-related arousal (Sinha et al. 2009). It is important to note that these alterations cannot be accounted for by smoking status or lifetime history of anxiety or mood disorders and therefore seem to be related to history of chronic alcohol abuse. The persistence of emotional distress and alcohol craving induced by stress and alcohol cue exposure suggests a dysfunction in emotion regulatory mechanisms. As HPA axis responses and autonomic–parasympathetic responses contribute to regulating and normalizing stress responses and regaining homeostasis, dysfunction in these pathways and their related central mechanisms may be involved in perpetuating alcohol craving and relapse susceptibility.

## Laboratory Response to Relapse Situations and Subsequent Alcohol Relapse

An important aspect of modeling hallmark addictive symptoms, such as alcohol craving, in the laboratory is to understand the related mechanisms. Furthermore, researchers should test the predictive validity of the laboratory model by examining whether laboratory responses predict future drug-use behaviors and/or real-world clinical outcomes. Because the laboratory studies described earlier were conducted with treatment-engaged alcoholics who were inpatients at a treatment research unit, it was possible to assess relapse rates after discharge. Then researchers could examine specific markers of the stress and craving states that are predictive of relapse outcomes. They followed the alcohol-dependent individuals (who had been in inpatient treatment for 5 weeks) after discharge for 90 days to assess relapse outcomes. Face-to-face follow-up assessments were conducted at 14, 30, 90, and 180 days after discharge from the inpatient unit. The follow-up rates for these assessments were 96, 89, 92, and 86 percent, respectively.

Initial evidence suggested that laboratory responses to stress- and alcohol-related stimuli exposure were predictive of alcohol treatment outcomes. Stress-induced alcohol craving in the laboratory during inpatient treatment was predictive of number of days of alcohol used and total number of drinks consumed during the 90-day follow-up period (Breese et al. 2005). These data corroborate findings in cocaine abusers, showing that stress-induced cocaine craving and HPA arousal are associated with earlier relapse and more cocaine use at follow-up (Sinha et al. 2006). In a more comprehensive analysis of stress dysregulation, anxiety, alcohol craving, and subsequent return to drinking, researchers found clear evidence of stress dysregulation and alcohol craving relating to relapse risk (Sinha et al. 2011*a*). Alcohol-dependent patients, compared with the control group, were more likely to have significant HPA axis dysregulation, marked by higher basal ACTH and higher basal salivary cortisol, lack of stress- and cue-induced ACTH and cortisol responses, higher anxiety after exposure to neutral relaxed and to alcohol cues, and greater stress- and cue-induced alcohol craving (Sinha et al. 2009, 2011*a*). Stress- and cue-induced anxiety and stress-induced alcohol craving were associated with fewer days in aftercare alcohol treatment. High alcohol craving to both stress and to alcohol cue provocation and greater neutral-relaxed state cortisol/ACTH ratio (adrenal sensitivity) were each predictive of shorter time to alcohol relapse. Although a greater cortisol-to-ACTH ratio in the stress and alcohol cue conditions also predicted relapse, the strongest predictor of relapse was the neutral relaxed state adrenal sensitivity (Sinha et al. 2011*a*). These results identify a significant effect of high adrenal sensitivity, anxiety, and increased stress-and cue-induced alcohol craving on subsequent alcohol relapse and treatment outcomes. They also are consistent with earlier reports of stress system involvement in relapse outcomes in alcoholics. Negative mood and stress-induced alcohol craving and blunted stress and cue-induced cortisol responses have been associated with alcohol relapse outcomes (Breese et al. 2005; Cooney et al. 1997; Junghanns et al. 2003). In summary, these findings support the involvement of stress-related pathophysiology in the alcohol relapse process. Among alcoholics in early recovery, the alcohol-craving state is marked by anxiety and compulsive motivation for drugs, along with poor stress regulatory responses (i.e., high basal HPA axis responses but blunted stress HPA responses), resulting in an enhanced susceptibility to addiction relapse.

## Brain-Imaging Studies of Alcoholics’ Responses to Alcohol Cues and Stress and Implications for Relapse Risk

Several studies have used brain-imaging techniques to assess chronic alcohol-related brain changes and whether such changes are associated with alcohol craving and alcohol use. Neuroanatomically, the cortico–striatal–limbic brain regions have been most studied in the context of stress, emotion, and motivation for alcohol reward. These regions include the frontal and insular cortices, the ventral and dorsal striatum, the amygdala, hippocampus, and thalamic nuclei, and midbrain regions, such as the VTA and the substantia nigra. An early study to measure blood flow with single-photon emission computed tomography found a change in the caudate nucleus during induction of craving in alcoholics (Modell and Mountz 1995). Subsequently, George and colleagues (2001) found a greater increase in brain response to alcohol cues in alcoholics compared with controls in the anterior thalamus and left dorsal lateral prefrontal cortex using functional magnetic resonance imaging (fMRI). Using a memory task during fMRI, Tapert and colleagues (2001) found dysfunctional cortical responses in alcoholics distinct from those of control subjects. Subsequently, other imaging studies with alcoholic patients have shown an increased association between dorsal striatum regions and alcohol craving in response to the presentation of alcohol-related stimuli (Grusser et al. 2004; Wrase et al. 2002). Myrick and colleagues (2004) reported that alcohol cues produced changes in the left orbital frontal cortex, anterior cingulate cortex, and nucleus accumbens in alcoholics but not in other study participants (Myrick et al. 2004).

Using fMRI, Sinha and colleagues (2007) compared alcohol-dependent individuals abstinent from alcohol for 4 weeks with social drinkers to assess brain structural changes and also functional responses to stress, alcohol cues, and neutral relaxing guided imagery. Alcoholic patients showed greater activity in the ventromedial prefrontal cortex, the ventral striatum, insula, and specific regions of the thalamus and cerebellum during the neutral-relaxing condition (Sinha 2007; Sinha and Li 2007). These findings indicate that abstinent alcoholics show overall hyper-responsivity of the medial prefrontal and striatal-limbic regions, with no differences in brain responses to the neutral relaxed and stressful cues (Sinha and Li 2007; Sinha et al. 2007*a*). Hyperresponsivity of prefrontal and striatal-limbic regions is consistent with an overall kindling^2^ process, which blunts the neural informational processing responses to stressful stimuli, resulting in a dysregulated response to stress in alcoholics (see also review by Breese et al. 2011).

Using positron emission tomography (PET) techniques, researchers have documented reduced glucose metabolism, especially in frontal regions during both acute and protracted alcohol withdrawal (up to 3 to 4 months) (see Volkow and Fowler 2000 for review). Alcoholics also show significant reductions in dopamine D_2_ receptors compared with nonalcoholics, particularly in frontal-striatal regions (Volkow and Fowler 2000). Researchers have reported significant associations between dopamine D_2_ receptor binding in the ventral striatum and alcohol craving (Heinz et al. 2004, 2005) as well as motivation for alcohol self-administration in alcoholics (Martinez et al. 2005, 2007). To emphasize the importance of this approach, recent PET studies have shown significant positive correlations between selected dorsal striatum brain regions and drug cue–induced cocaine craving (Volkow et al. 2006; Wong et al. 2006). These data point to alterations in frontal and striatal regions of the dopaminergic and noradrenergic pathways that exist past acute withdrawal and may be associated with difficulties in regulating emotions, stress, and problems selecting goal-directed adaptive responses as opposed to the selection of habitual maladaptive responses such as alcohol consumption.

In addition, the research literature has documented chronic alcohol-related structural brain changes, particularly in frontal, parietal, and temporal cortical regions associated with stress, emotion, and cognitive functioning (Cardenas et al. 2007; Fein et al. 2002; Pfefferbaum et al. 1995, 1998). More severe gray matter deficits have been reported in alcohol relapsers than those who maintained abstinence (Pfefferbaum et al. 1998). In a whole-brain analysis, Rando and colleagues (2011) found significantly smaller gray-matter volume in recently abstinent alcohol-dependent patients relative to healthy study participants in three regions: the medial frontal cortex, right lateral prefrontal cortex, and a posterior region surrounding the parietal-occipital sulcus. Smaller medial frontal and parietal-occipital gray-matter volume were each predictive of shorter time to subsequent any alcohol use (first lapse) and to heavy-drinking relapse (Rando et al. 2011). These data suggest that smaller gray-matter volume in specific medial frontal and posterior parietal-occipital brain regions are predictive of an earlier return to alcohol drinking and relapse risk, suggesting a significant role for gray matter atrophy in poor clinical outcomes in alcoholism. Thus, the extent of gray-matter volume deficits in these regions involved in impulse control, emotion regulation, and abstraction abilities could serve as useful neural markers of relapse risk and alcoholism treatment outcome.

## Clinical Implications and Conclusion

The previous sections cite evidence from clinical, laboratory, and neuroimaging studies to examine whether stress increases the risk of relapse. Psychobiological and neuroimaging research points to alcohol-related changes in brain volume and function and in biological stress responses. These alterations were found to contribute to higher craving and increased alcohol relapse risk. For example, early abstinence from alcohol is associated with higher levels of anxiety when relaxed and when exposed to alcohol cues, greater emotional distress, and increased stress- and alcohol cue-induced craving. These states are accompanied by disruption in normal functioning of the peripheral stress pathways, including the HPA axis and the autonomic components, which are involved in mobilizing the body for action during stress but also in physiological regulation of the stress response. A lack of normal stress regulation during this early abstinence period leaves the recovering alcoholic highly vulnerable to high craving, anxiety, and risk of relapse, particularly under stressful conditions and when faced with alcohol-related stimuli in the environment. The findings discussed indicate that stress- and cue-induced alcohol craving increase the risk of subsequent relapse. High levels of stress- and cue-induced anxiety are associated with less follow-up in aftercare during the recovery period. Furthermore, disrupted functioning of the HPA axis, particularly in people who have hyperresponsive cortisol release from the adrenal cortex in response to the ACTH signal (cortisol-to-ACTH ratio as a measure of adrenal sensitivity) in the neutral relaxed state, increased the risk of alcohol relapse 2.5 times more than those with lower cortisol release from the adrenal cortex. Finally, changes in volume and function of the brain regions involved in impulse control and emotion regulation also are predictive of alcohol relapse outcomes. Each of these measures could be further developed as biomarkers of alcohol relapse risk (see Sinha 2011). If validated in future studies, they may be used clinically to identify people at high risk of relapse. In addition, the findings reviewed also indicate that stress-related pathophysiology is important in the alcohol relapse process. Thus, individuals who show chronic alcohol-related effects on neural, biological, and psychological aspects of stress and craving could benefit from treatments that target stress effects on craving and alcohol seeking. Several novel medications that target the stress pathways, such as agents that block CRF, as well as noradrenergic and GABAergic agents, are being tested to assess their efficacy in stress-related relapse (Breese et al. 2011; Sinha et al. 2011*b*). Development of such treatment strategies may be of tremendous help in normalizing stress responses and decreasing alcohol craving so as to improve relapse outcomes in alcoholism.

## Footnotes

1 For definitions of this term and other technical terms used in this article, see the Glossary on pp. 522–524.

2 Kindling is a phenomenon in which a weak electrical or chemical stimulus, which initially causes no overt behavioral responses, results in the appearance of behavioral effects, such as seizures, when it is administered repeatedly.
